# Electro‐acupuncture and its combination with adult stem cell transplantation for spinal cord injury treatment: A summary of current laboratory findings and a review of literature

**DOI:** 10.1111/cns.13813

**Published:** 2022-02-17

**Authors:** Yuan‐Shan Zeng, Ying Ding, Hao‐Yu Xu, Xiang Zeng, Bi‐Qin Lai, Ge Li, Yuan‐Huan Ma

**Affiliations:** ^1^ Key Laboratory for Stem Cells and Tissue Engineering (Sun Yat‐sen University) Ministry of Education Guangzhou China; ^2^ Department of Histology and Embryology Zhongshan School of Medicine Sun Yat‐sen University Guangzhou Guangdong Province China; ^3^ Guangdong Provincial Key Laboratory of Brain Function and Disease Guangzhou Guangdong Province China; ^4^ Co‐innovation Center of Neuroregeneration Nantong University Nantong China; ^5^ Institute of Spinal Cord Injury Sun Yat‐sen University Guangzhou Guangdong Province China

**Keywords:** bone marrow mesenchymal stem cells, electrical stimulation, electro‐acupuncture, neuromodulation, neurotrophic factors, receptor tyrosine kinases; neural stem cells, spinal cord injury

## Abstract

The incidence and disability rate of spinal cord injury (SCI) worldwide are high, imposing a heavy burden on patients. Considerable research efforts have been directed toward identifying new strategies to effectively treat SCI. Governor Vessel electro‐acupuncture (GV‐EA), used in traditional Chinese medicine, combines acupuncture with modern electrical stimulation. It has been shown to improve the microenvironment of injured spinal cord (SC) by increasing levels of endogenous neurotrophic factors and reducing inflammation, thereby protecting injured neurons and promoting myelination. In addition, axons extending from transplanted stem cell‐derived neurons can potentially bridge the two severed ends of tissues in a transected SC to rebuild neuronal circuits and restore motor and sensory functions. However, every single treatment approach to severe SCI has proven unsatisfactory. Combining different treatments—for example, electro‐acupuncture (EA) with adult stem cell transplantation—appears to be a more promising strategy. In this review, we have summarized the recent progress over the past two decades by our team especially in the use of GV‐EA for the repair of SCI. By this strategy, we have shown that EA can stimulate the nerve endings of the meningeal branch. This would elicit the dorsal root ganglion neurons to secrete excess amounts of calcitonin gene‐related peptide centrally in the SC. The neuropeptide then activates the local cells to secrete neurotrophin‐3 (NT‐3), which mediates the survival and differentiation of donor stem cells overexpressing the NT‐3 receptor, at the injury/graft site of the SC. Increased local production of NT‐3 facilitates reconstruction of host neural tissue such as nerve fiber regeneration and myelination. All this events in sequence would ultimately strengthen the cortical motor‐evoked potentials and restore the motor function of paralyzed limbs. The information presented herein provides a basis for future studies on the clinical application of GV‐EA and adult stem cell transplantation for the treatment of SCI.

## Graphical abstract

A schematic diagram showing that EA stimulates the peripheral nerve endings of the meningeal branch, which transmits information to the SC through nerve fibers of neurons in the DRG. EA can promote the survival and differentiation of transplanted stem cell‐derived neurons or neuron‐like cells in injured SC by inducing the secretion of neurotrophin‐3 by host neurons, which promotes their integration into existing neural circuits.

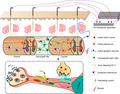

## 1 INTRODUCTION

There is presently no effective treatment for severe spinal cord injury (SCI) because the microenvironment of the injured spinal cord (SC) tissue is not conducive to self‐repair of disrupted neural pathways.^1^ As such, improving the microenvironment of injured SC tissue, so that it promotes tissue repair, has been the focus of intense research.^2^ Neurotrophic factors that have been used for this purpose include nerve growth factor (NGF), brain‐derived neurotrophic factor (BDNF), and neurotrophin (NT‐3).^3^

Both NGF and its receptor, tyrosine kinase (Trk)A, mainly act on sensory neurons,^4^ with no obvious effects on motor neurons. BDNF and its receptor, TrkB, act on a limited number of neuron types.^5^ NT‐3 and its receptor, TrkC, play an important role in the development and differentiation of neurons, as well as in the survival and axonal regeneration of damaged central neurons (in the brain and SC).^6^ Furthermore, NT‐3 has also been shown to promote the regeneration of corticospinal tract nerve fibers after SCI,^7^ although the effect is not maintained over a long period. To address this problem, our research team has adopted a neuromodulation technique, based on traditional Chinese medicine acupuncture combined with modern electrical stimulation (EA) to trigger the continuous secretion of endogenous NT‐3 in the SC.

In traditional Chinese medicine, paraplegia caused by SCI is categorized as an “indolence,” “wind,” and “sputum” syndrome and is thought to result from damage to the Governor Vessel (GV),^8^ which regulates the function of all yang medians. SCI is associated with GV damage, which prevents the flow of qi and blood, leading to atrophy and loss of muscle function.^9^ In theory, EA at the GV acts directly on a diseased area such as the injured SC to replenish the yang medians and increase their accessibility, thereby alleviating paraplegia. Moreover, EA at the GV acupoints (located at the posterior midline of the trunk) is the preferred treatment for paraplegia caused by SCI.^8^, ^9^, ^10^, ^11^, ^12^

A key concept of GV‐EA is that stimulation at specific somatic tissues/acupoints can modulate internal SC physiology. In GV‐EA, a small low‐frequency pulsed current is delivered through needles inserted at the GV acupoints, which ventilates the meridians to promote qi and blood flow.^8^ Thus, this therapy has the dual effects of acupuncture and electrical stimulation. Furthermore, GV‐EA has been shown to improve traumatic paraplegia.^13^ Some aspects of the mechanism underlying this effect have been elucidated over the last two decades. Both EA and GV‐EA can alleviate secondary damage after SCI, protect neurons, and stimulate nerve fiber regeneration and functional recovery by regulating the levels of neurotrophic factors, neurotransmitters, neuropeptides, and intracellular signaling pathway components.^14^, ^15^, ^16^, ^17^

Our previous work has demonstrated that GV‐EA improves the microenvironment of the injured SC and promotes the regeneration of damaged nerve fibers and functional recovery.^18^ EA was shown to stimulate the migration, proliferation, and differentiation of endogenous neural stem cells (NSCs) at the injury site in the SC of rats.^19^, ^20^, ^21^ Under normal conditions, these NSCs are induced to differentiate into astrocytes by factors in the microenvironment^22^ that participate in the formation of glial scars for tissue stabilization and repair. NSCs rarely differentiate into neurons to replace those that have died due to injury; therefore, whereas GV‐EA can promote SC repair, the depletion of endogenous NSCs, or their insufficient differentiation into neurons, prevents the restoration of neuronal circuits in the absence of any transplantation of exogenous NSCs.^23^

The transplantation of NSCs into the injured SC is a cell‐based therapy that has been used for more than two decades.^1^ When embryonic neural tissue is transplanted into the developing or adult SC, NSCs or newborn neurons replace lost nerve cells, contributing to the reconstruction of neuronal circuits and functional recovery.^24^ NSCs can be induced to differentiate into neurons, astrocytes, and oligodendrocytes under appropriate conditions. NSCs for transplantation are mainly derived from embryonic and adult tissues. Adult NSCs repair SC injury by replacing dead neurons and thus facilitate the reconstruction of neuronal circuits^25^; the myelination of regenerated nerve fibers,^26^ and secreting neurotrophic factors to improve the microenvironment of the injured SC and promote nerve fiber regeneration.^27^

Bone marrow mesenchymal stem cells (MSCs)—another type of adult stem cell that can be isolated from a variety of sources—rapidly proliferate in vitro and exhibit multidirectional differentiation potential. When transplanted into the body, MSCs can be induced to differentiate into chondroblasts, osteoblasts, adipocytes, myoblasts, cardiomyocytes, hepatocytes, oligodendrocytes, and neuron‐like cells by factors in the local microenvironment.^28^, ^29^ As there are no ethical constraints to autologous transplantation of MSCs and no risk of immunologic rejection, MSCs can serve as substrates for cell and gene therapy in a variety of diseases^1^ and represent a potential cellular source for SCI treatment. However, only a small number of adult NSCs differentiate into neurons following direct transplantation into the injury/graft site of the SC, whereas grafted adult MSCs are less likely to differentiate into neuron‐like cells, and therefore cannot replace dead neurons. The efficiency of cell‐based therapy for SCI can potentially be enhanced by other treatment strategies such as EA.

It is apparent from the literature that there are some critical issues and inherent limitations that need to be fully addressed whether we are to treat SCI more effectively. We consider the following strategies crucial for the better treatment of SCI: (1) to improve the microenvironment of the damaged tissue so it is conducive for the enhancement of nerve regeneration after SCI; (2) to replace the dead neurons, oligodendrocytes, and other major functional cells, and (3) to promote the better integration of replacement functional cells into the original neural network of the SC. Collectively, these factors will eventually help to repair the damaged structure and restore function in the SC. In this review, we summarize the research conducted over the last two decades relating to the therapeutic potential of GV‐EA in combination with adult stem cell transplantation for the repair of the injured SC, paying particular focus upon the data generated by our laboratory.

## 2 GV‐EA PROMOTES THE SURVIVAL OF INJURED SC NEURONS

The application of acupuncture and EA for the treatment of nervous system disorders has become widely accepted over recent years, particularly as the mechanistic basis of their effects have been elucidated.^30^ Recent research revealed that the neuroprotection provided by EA was associated with activation of the parasympathetic nervous system in experimental stroke model animals.^31^ EA pretreatment also can increase ambient endocannabinoid levels and result in activation of the ischemic penumbral astroglial cannabinoid type 1 receptor, which led to moderate upregulation of extracellular glutamate that protected neurons from cerebral ischemic injury.^32^ In addition, EA can promote the survival and synaptic plasticity of hippocampal neurons and improve spatial memory disorders caused by sleep deprivation.^33^ Interestingly, a previous research suggested that EA protected cerebral hippocampal neurons in vascular dementia by inhibiting the expression of p53 (a tumor suppressor) and Noxa (p53 downstream effector) in hippocampal CA1 region.^34^ In response to EA, neurons synthesize and secrete specific proteins and neuropeptides that transduce the electrical signals. At different frequencies, EA can reportedly cause the central nervous system to release different types of neuropeptides that promote tissue recovery.^35^ Neuropeptides are a class of neurotransmitters or neuromodulators that are characterized by slow induction and prolonged action in a manner that is comparable to the slow conduction of EA needles.

The SC receives direct peripheral sensory input from the trunk and limbs, with peptidergic nerve fibers projecting to the dorsal horn. The fiber endings localized at the acupoints sense the needle and then release peptide substances (e.g., substance P [SP], vasoactive intestinal peptide [VIP], and neuropeptide [NP]Y).^36^ Research suggests that GV‐EA causes cells in the damaged SC to secrete acupoint‐specific proteins and neuropeptides that promote neuronal survival. In our laboratory, we found that GV‐EA increases the expression of acupoint‐specific proteins, annexin (ANX)A5, and collapsin response‐mediated protein (CRMP)2 in the injured SC tissue of rats.^16^ Moreover, the application of EA at non‐acupoints, and at the Huatuo‐jiaji acupoint, has been shown to downregulate these proteins.

In addition, ANXA5 and CRMP2 have been shown to exert protective effects on injured neurons in the SC nucleus dorsalis.^16^ In other studies, we showed that GV‐EA, EA at non‐acupoints, and EA at the Huatuo‐jiaji acupoint, has distinct effects on calcitonin gene‐related peptide (CGRP) levels in the injured SC tissue of rats; the application of GV‐EA increased these expression levels.^37^, ^38^ Moreover, CGRP promotes the survival of injured neurons in the nucleus dorsalis and red nucleus of the midbrain. At the Huatuo‐jiaji acupoint, GV‐EA was also found to be more effective than EA in reducing the expression of nitric oxide synthase in injured neurons of the nucleus dorsalis, thus enhancing neuronal survival.^39^ These findings provide evidence that EA can stimulate afferent nerve fibers in GV acupoints to induce the synthesis and secretion of specific proteins or neuropeptides by SC neurons to promote the survival of injured neurons.

## 3 GV‐EA STIMULATES THE SC VIA AFFERENT NERVE FIBERS OF THE MENINGEAL BRANCH

**FIGURE 1:**
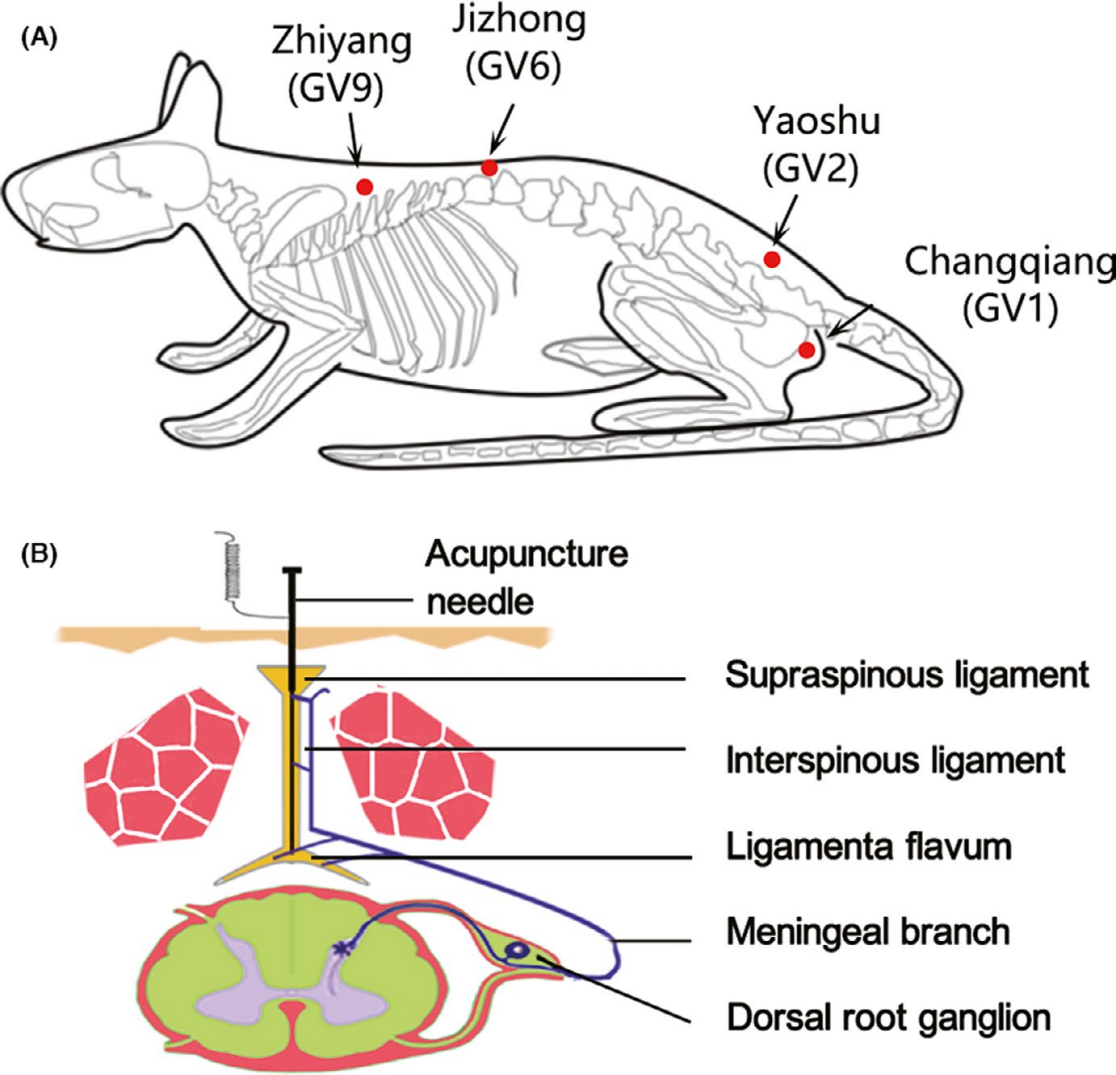
Showing two pattern diagrams of the location of the GV‐EA, taken from Yang Yang et al., CNS Neurosci Ther (2021).^93^ (A) A pattern diagram indicates selected electro‐acupuncture acupoints located at GV in rat. Arrows show the four GV acupoints: Zhiyang (GV9), Jizhong (GV6), Yaoshu (GV2), and Changqiang (GV1). (B) Another pattern diagram of the afferent nerve pathway of GV‐EA stimulation and the figure indicates that the stimulation information of GV‐EA might have been transmitted first to the dorsal root ganglion (DRG) by peripheral branches of meningeal branch and finally transmitted to the spinal cord by the DRG’s central branches

The use of GV‐EA for the treatment of SCI in rats is generally performed at acupuncture points below the spinous processes of the vertebral column corresponding to the upper and lower segments of the injury site (at an insertion depth of about 5 mm that is achieved by twirling, Figure 1). The positive and negative electrodes deliver a low‐frequency electrical pulse through upper and lower 0.30‐mm diameter needles.^37^ In our previous work, stimulation was performed with a sparse‐dense wave alternating stimulation pulse (alternating between 50 Hz/1.05 s and 2 Hz/2.85 s, with a pulse width of 0.5 ms), at a current intensity of approximately 5 µA between the two acupoints flanking the lesion site (the intensity was determined based on the observation of a slight vibration of the rat's muscle); the retention time was 20 min, and the frequency was once every other day.^40^

In our previous studies, we speculated that GV‐EA acts directly on the meningeal branch and perhaps also the dorsal (posterior) ramus of the spinal nerve of the SC. The acupuncture needle that penetrates the acupoint should pass through the skin, supraspinous ligament, interspinous ligament, and ligamentum flavum. It has been reported that the posterior longitudinal ligament of the vertebral bodies is also innervated by nerve endings of the meningeal branch.^41^ The meningeal branch contains the sensory afferent nerve fibers from the dorsal root ganglion (DRG) and some sympathetic nerve fibers originating from the adjacent paravertebral ganglia.^41^ We hypothesized that electrical stimulation would activate the GV acupoint to produce local sensory signals that are conveyed to the SC through afferent nerve fibers.^40^

**FIGURE 2:**
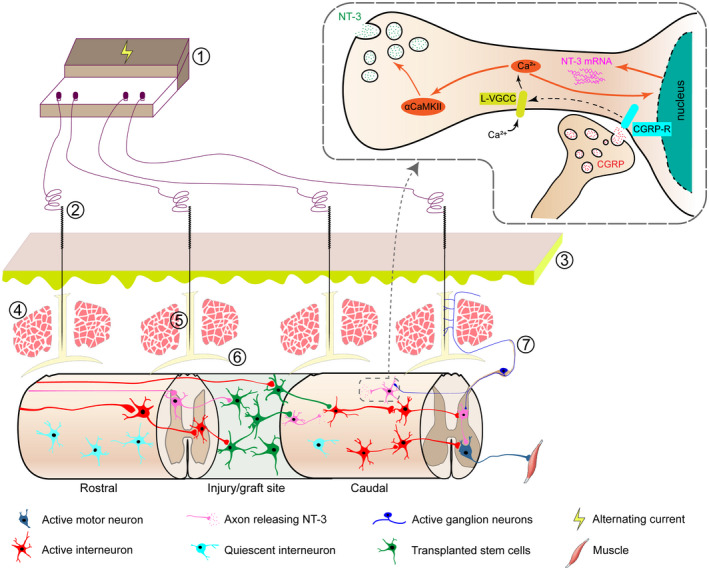
Schematic illustration of Governor Vessel electro‐acupuncture (GV‐EA) combined with stem cell transplantation for spinal cord injury (SCI) repair. ① EA therapeutic apparatus; ② acupuncture needle; ③ skin; ④ lumbus muscle; ⑤ supraspinous ligament, interspinous ligament, ligamenta flavum, and vertebral periosteum; ⑥ spinal dura mater; ⑦ meningeal branch

To test this hypothesis, we injected red fluorescent cholera toxin B subunit CTB‐555 into the GV acupoint region to mark afferent nerve fibers of the meningeal branch entering the SC across the DRG. Subsequently, we observed CTB‐555 into the GV acupoint region to mark afferent nerve fibers of the meningeal branch entering the SC across the DRG. CTB‐555 labeling was observed along the needle trajectory and in nerve fibers of the meningeal branch in the posterior longitudinal ligaments of the vertebrae. Moreover, CTB‐555‐labeled sensory neurons were observed in the DRG, and sensory afferents labeled with red fluorescence were visible in the dorsal horn in corresponding segments.^40^ These results support the ability of afferent nerve fibers of the meningeal branch, which ramify in GV acupoints, to transmit EA stimulation to the SC (Figure 2).

GV‐EA increases the expression of CGRP in injured SC tissue; this may be attributable to electrical signals transmitted to the inside of the SC via the afferent nerve fibers of the meningeal branch of the SC.^37^ SC tissue does not itself synthesize CGRP, which is thought to be mainly produced by DRG sensory neurons in the corresponding spinal segments and transmitted to the dorsal horn of the SC through afferent nerve fibers.^42^ To investigate this possibility, we performed unilateral dorsal root transection combined with lidocaine injection at GV acupoints to block the local delivery of information by afferent nerve fibers in the meningeal branch of the SC. This strategy resulted in the downregulation of CGRP in the injured SC segment. Next, we speculated that electrical stimulation from GV‐EA might be able to reach injury sites in the SC *via* afferent nerve fibers of the meningeal branch of the spinal nerve that are distributed at the GV acupoint. Subsequently, we demonstrated unequivocally that GV‐EA can induce the upregulation of CGRP in the DRG cells and its subsequent release at the injury site of SC.^37^, ^40^ Collectively, these data indicate that the electrical signals generated by GV‐EA stimulation are transmitted through afferent nerve fibers in the meningeal branches to the relevant spinal cord segment, in which their nerve endings secrete a neuropeptide (CGRP) to exert neurobiological effect.

## 4 GV‐EA INDUCES THE SYNTHESIS AND SECRETION OF NEUROTROPHIC FACTORS BY CELLS IN THE INJURED SC

Following SC transection and demyelination, cells in the injured area and adjacent tissue undergo a number of pathophysiological changes, including the reduced synthesis and secretion of neurotrophic factors; this is one of the main factors underlying the inhibition of nerve repair. An important therapeutic mechanism of GV‐EA in SCI is to induce cells in the SC to synthesize and secrete endogenous neurotrophic factors,^43^ which creates a microenvironment that is conducive to neuronal survival and axonal regeneration.^14^, ^44^, ^45^

NT‐3 is a neurotrophic factor that plays an important role in preventing the death of injured neurons, enhancing neuronal survival and axonal regeneration, and inducing endogenous oligodendrocyte precursor cells to differentiate into mature oligodendrocytes that can restore the myelin sheath.^46^ GV‐EA has been shown to stimulate the synthesis and secretion of NT‐3 by various cell types in transected SC tissue,^47^, ^48^, ^49^ including neurons, astrocytes, microglia/macrophages, and oligodendrocytes. The secretion of NT‐3 by these cells progressively increases from Day 1 through 7 of GV‐EA application; the level on Day 7 is significantly higher than that prior to the application of EA..^47^ We also found that NT‐3 levels were significantly higher in the EA group than in the control group on Days 14 and 28.^48^, ^49^ Other studies have confirmed that GV‐EA can increase the synthesis and secretion of NT‐3 in the demyelinated SC tissue of rats.^50^, ^51^, ^52^

GV‐EA increases the expression of endogenous NT‐3 in the SC, activates NT‐3/TrkC/protein kinase B (AKT) signaling, and enhances TrkC‐overexpressing NSC‐derived neurons of transplanted neural network tissue to promote the establishment of synaptic connections with host neurons that secrete NT‐3.^53^ However, the mechanisms underlying these effects are unclear. GV‐EA induces the depolarization of neurons,^54^, ^55^ thus leading to the opening of L‐type voltage‐gated Ca^2+^ channels (L‐VGCC) and Ca^2+^ influx; this process activates calcium/calmodulin‐dependent protein kinase (αCaMKII) signaling and the synthesis and secretion of neurotrophic factor (e.g., NT‐3).^55^

Thus, GV‐EA may directly stimulate the afferent nerve fiber endings of the meningeal branch of the SC in adjacent segments and induce the release of CGRP by DRG neurons into the SC. CGRP acts on spinal neurons expressing receptor activity‐modifying protein (RAMP)1, causing the opening of L‐VGCC, and ultimately leading to the release of NT‐3.^40^ There is increasing evidence that epidural electrical stimulation or transcutaneous SC stimulation promotes the synthesis and secretion of neurotrophic factors in the injured SC, which improves the local microenvironment and activates neural circuits in the SC^56^, ^57^ through mechanisms similar to those of GV‐EA.

It is important to note that following GV‐EA, NT‐3 is not just produced by neurons in the SC; there is evidence that endogenous NT‐3 is also produced by the astrocytes. NT‐3, along with microglia and macrophages may also contribute to the restoration of damaged SC tissue. NT‐3, synthesized and secreted by reactive astrocytes in the rostral and caudal host tissues adjacent to the injury/graft site of SC, may protect the damaged neurons and facilitate axonal regeneration of transplanted NSC‐derived neurons or MSC‐derived neuron‐like cells and host neurons.^46^, ^58^, ^59^ In addition, NT‐3 secreted by activated microglia/macrophages may induce the polarization of proinflammatory M1‐type microglia/macrophages to anti‐inflammatory repair M2‐type microglia/macrophages, thus reducing the inflammatory response.^60^, ^61^, ^62^, ^63^ However, the exact mechanisms by which NT‐3 inhibits the inflammatory response for SCI repair have yet to be elucidated.

## 5 GV‐EA PROMOTES NT‐3 SECRETION IN THE INJURED SC TO ALLEVIATE MUSCLE ATROPHY IN PARALYZED LIMBS

Studies have shown that sensory and motor functions are lost below the level of injury following severe SCI.^64^ Due to the loss of innervation, the volume and weight of the muscle in the paralyzed limbs are reduced as the muscles undergo atrophy,^65^, ^66^ thus further hindering the recovery of motor function. Therefore, maintaining muscle function is essential for functional recovery from SC injury.^67^ Although the ascending and descending neural pathways are transected in a thoracic segment SCI, below the injury level, the structural basis of the neural circuits that regulate the lower limbs remain in the lumbar segment of the SC.^68^, ^69^, ^70^ These neural circuits may be functionally silent, and their reactivation can promote the nerve‐muscle connection and prevent muscle atrophy.^65^, ^66^, ^67^, ^68^, ^69^, ^70^ We applied GV‐EA to rats with a completely transected SC in the thoracic segment and found that muscle atrophy in the paralyzed hindlimbs was improved, which was associated with increased survival of motor neurons and NT‐3 expression in the lumbar SC.^71^ This may be attributed to the transmission of electrical stimulation to the SC segment through primary afferent nerve fibers.^40^, ^66^, ^72^

NT‐3 also plays an important role in neuroprotection, axon regeneration, and synaptic plasticity.^73^, ^74^ The effects of GV‐EA on the survival of motor neurons in the lumbar SC are speculated to be related to an increase in NT‐3 levels.^71^ GV‐EA also stimulates the release of neurotransmitters (such as CGRP and acetylcholine, among others) in the lumbar SC, which enhances the excitability of specific neuronal circuits that lead to the contraction of target muscles innervated by the surviving motor neurons, thereby alleviating muscle atrophy.^40^, ^71^ Based on the above results, it is reasonable to suggest that electrical stimulation can improve muscle atrophy in paralyzed limbs in the early stages of SCI, laying the foundation for the next step of adult tissue stem cell transplantation to enhance the voluntary motor function of paralyzed limbs.

## 6 GV‐EA ALLEVIATES SCI BY PROMOTING THE INTEGRATION OF TRANSPLANTED STEM CELLS

Approximately half of all SCIs involve complete SC transection, with little or no remaining nerve tissue in the injured segment. Restoration of SC tissue—for example, through cell transplantation—is essential for the effective treatment of SCI.^1^, ^75^ NSCs or MSCs transplanted into the injured SC are induced to differentiate into neurons or neuron‐like cells and glial cells that restore SC structure and function. Transplanted NSCs have been shown to differentiate into neurons and glia, thereby reducing secondary damage to the SC and improving spinal motor function.^76^ However, they are also more likely to differentiate into astrocytes than neurons, which is a major barrier to the clinical application of cell‐based therapies for SCI.

One way to overcome this problem is to use GV‐EA in combination with NSC transplantation to treat SCI.^77^ GV‐EA has been shown to promote the survival, migration, and neuronal differentiation of NSCs transplanted into the injury/graft site of the SC.^77^ This strategy can also enhance the survival of injured host neurons and nerve fiber regeneration, as well as evoked potentials in the cerebral cortex motor; these processes help to reduce muscle atrophy and immobility in paralyzed limbs.^49^, ^78^, ^79^ These effects can be attributed to the GV‐EA‐induced stimulation of NT‐3 secretion by cells in the injured SC tissue.

The NT‐3‐induced activation of intracellular TrkC/AKT signaling is known to promote the differentiation of NSCs into neurons; this process can be blocked by the application of TrkC/AKT inhibitors.^80^ Thus, GV‐EA can exert multiple therapeutic effects in SCI, including improving the local microenvironment, increasing endogenous levels of NT‐3, activating TrkC/AKT signaling, enhancing the survival and differentiation of neurons, and promoting synapse‐like junction formation by transplanted NSCs, as well as their integration in spinal neuronal circuits; collectively, these processes lead to the restoration of sensory and motor functions.^53^

However, it is important to consider that there are key ethical barriers to NSC transplantation that limit its translational potential. In clinical studies of spinal cord treatment, the embryonic neural stem cells (NSCs) that are used are generally derived from human embryonic brain tissue or human embryonic spinal cord tissue. The ethical debate over whether embryonic NSC technology is “life‐damaging” was triggered by the destruction of embryos caused by selective abortion.^81^ However, most researchers agree that the process of consent for human embryonic neural stem cell research, and its clinical application, is fully informed and voluntary.^82^ Due to the fact that this is still an area associated with significant debate, the application of neural stem cells may be limited to translational clinical research for the time being. In contrast, there is no risk of immunological rejection or ethical issues associated with the transplantation of autologous MSCs; this process has been used successfully for the repair of bone, cartilage, tendon, and muscle tissues.^83^ Thus, MSC transplantation may also be used to repair injured SC.^84^, ^85^ A previous *in vitro* study demonstrated that NT‐3 induces MSCs to overexpress the NT‐3 receptor (TrkC) to differentiate into neuron‐like cells.^48^, ^86^ Within the last 10 years, studies in our laboratory, and others, demonstrated that the combination of EA and MSC transplantation provides a greater advantage than either procedure alone when applied to animals with SCI.^77^, ^87^, ^88^, ^89^, ^90^

**FIGURE 3:**
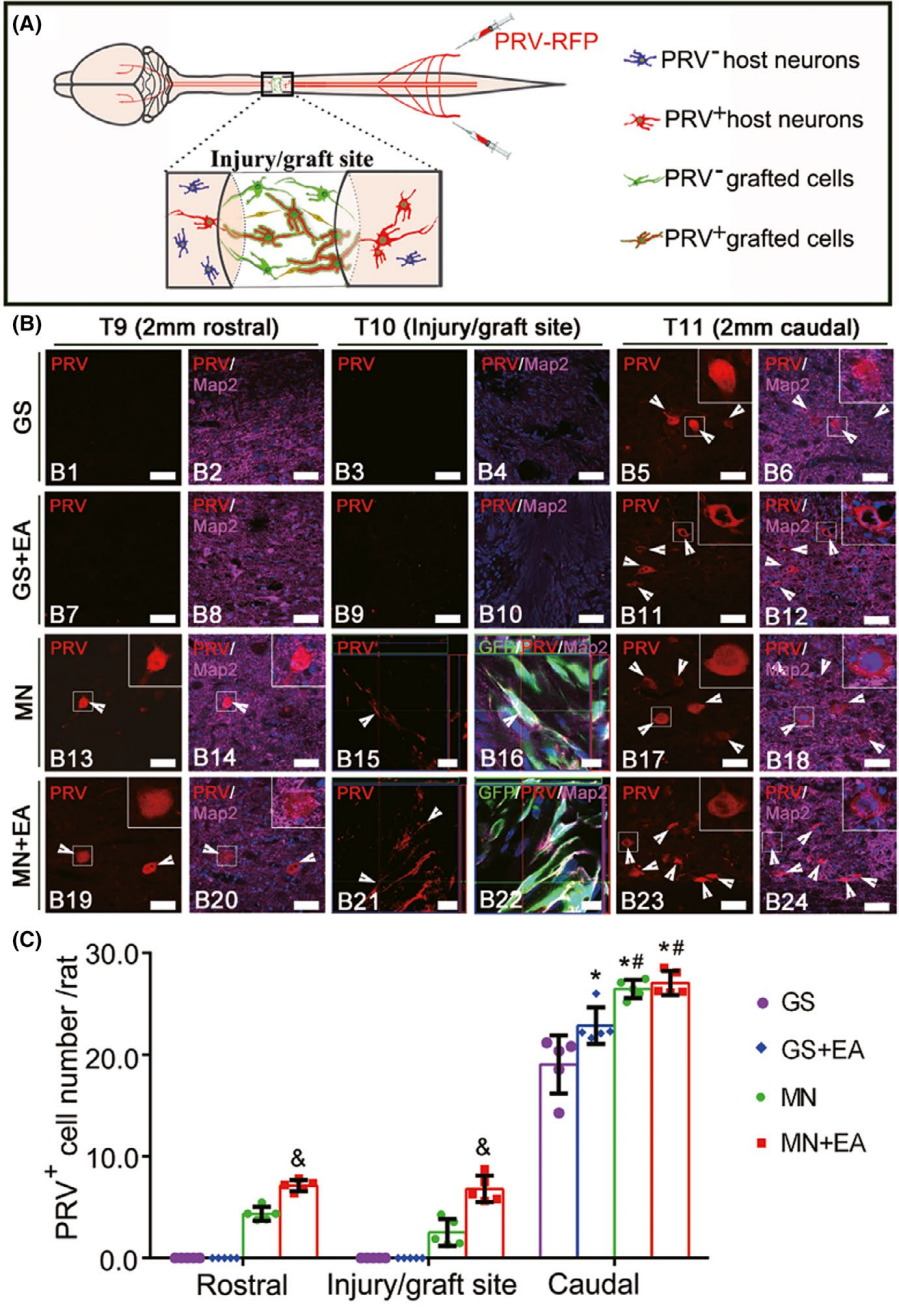
Pseudorabies virus (PRV) retrograde transsynaptic labeling confirmed the integration of transplanted mesenchymal stem cell (MSC)‐derived neuron‐like cells into the host spinal cord neuronal circuit, taken from Yang Yang et al.,^93^ CNS Neurosci Ther (2021). (A) A schematic diagram showing that PRV that was injected into the sciatic nerve was transported from the caudal area to the rostral area through the injury/graft site of the spinal cord. (B) Representative images showing the host neurons or MSC‐derived neuron‐like cells retrogradely labeled with PRV (red, arrowheads) in the rostral and caudal regions relative to the graft tissue of spinal cord in the gelatin sponge scaffold (GS) group (B1–B6), GS+EA group (B7–B12), MSC‐derived neural network (MN) group (B13–B18), and MN+EA group (B19–B24). The cell nuclei were counterstained with Hoechst33342 (Hoe). (C) Bar chart showing the number of PRV^+^ neurons in the T9, T10, and T11 areas of the 4 groups. Values represent the mean ± SD. *n* = 5/group. *p < 0.05, compared with the GS group, ^#^ *p* < 0.05, compared with the GS+EA group, and ^&^ *p* < 0.05, compared with the MN group by one‐way ANOVA with LSD‐t. Green fluorescent protein (GFP, green), PRV (red), microtubule‐associated protein (Map2, white), and Hoe (blue). Scale bars =50 µm in (B1)–(B14), (B17)–(B20), (B23), and (B24); 10 µm in (B15) and (B16), (B21), and (B22). GS: gelatin sponge scaffold with no cells; GS+EA: GS combined electro‐acupuncture; MN: MSC‐derived neural network; MN+EA: MN combined with electro‐acupuncture

Based on this observation, we used a combination of GV‐EA and the transplantation of *TrkC* gene‐modified MSCs to treat SCI.^48^, ^87^, ^91^, ^92^ This led to increased levels of NT‐3 expression at the injury/graft site in the SC and adjacent tissue; enhanced survival of neuron‐like and oligodendrocyte‐like cells differentiated from TrkC‐overexpressing MSCs; a decreased number of glial fibrillary acidic protein (GFAP)‐positive astrocytes; the reduced accumulation of the nerve regeneration inhibitory factor, chondroitin sulfate proteoglycan (CSPG) in the extracellular matrix; the increased accumulation of the nerve regeneration‐promoting factor laminin; an increased number of CGRP‐positive nerve fibers (ascending), as well as 5‐hydroxytryptamine (5‐HT)‐positive and corticospinal tract nerve fibers (both descending), and the formation of synapse‐like junctions; and strengthened cortical motor‐evoked potentials. Previous researches have showed that the MSC‐derived neuron‐like cells in the injury/graft site of spinal cord were integrated into the host neural circuitry; therefore, they can receive neural input from the rostral and caudal areas of the injury/graft site in the transected SC and transmit the information to host neurons on both sides of the gap (Figure 3).^3^, ^93^ As reported previously, the aim of the this particular strategy is to induce the transdifferentiation of *TrkC* gene‐modified MSCs into neuron‐like cells that can serve as a relay mechanism upon transplantation into a gap in the SC.^94^, ^95^, ^96^ To sum up, these processes promoted the regeneration of SC tissue and improved motor function in paralyzed limbs.

Although these transdifferentiated cells are neuron‐like, they express most neuron markers including cytoskeleton proteins (neurofilament [NF]‐200, NF‐H, microtubule‐associated protein [MAP]2, and β‐tubulin III); synaptic structure proteins (postsynaptic density [PSD]‐95 and synaptophysin); neurotransmitters (γ‐aminobutyric acid [GABA] and choline acetyltransferase [ChAT]); and doublecortin (DCX). We have also shown that some of the *TrkC* gene‐modified MSC‐derived cells exhibit electrophysiologic properties in vitro, such as postsynaptic currents and action potentials in the presence of Schwann cells overexpressing NT‐3.^93^, ^95^ Importantly, the cells form extensive connections or contacts with host neurons and descending 5‐HT‐positive nerve fibers. This unique cell type, with features of both neurons and MSCs (e.g., expression of hypoxia inducible factor [HIF]‐1α, prostaglandin [PG]‐E2, and vascular endothelial growth factor [VEGF]), may have specific roles in the repair of the injured SC.^95^ Nevertheless, this has yet to be demonstrated experimentally.

Collectively, these studies have shown that GV‐EA can improve the microenvironment surrounding the injury/graft area, enhance the survival rate of transplanted neural network tissue cell, and maintain their synaptic connections, thus promoting the functional integration of the donor tissue cells with the SC intrinsic neural circuit. Therefore, under the EA stimulation, the transplanted stem cell‐derived neural network tissue plays a role of preferably restoring SC structure and function as the neuronal relay in the injury/graft area.^97^

## 7 GV‐EA STIMULATES THE REPAIR BY TRANSPLANTED MSCS OF DEMYELINATION INJURY IN THE SC

Both mechanical and chemical SCI can lead to nerve fiber demyelination.^98^ We have previously shown that GV‐EA promotes the synthesis and secretion of NT‐3 in demyelinated areas of the SC and increases the number of endogenous oligodendrocyte precursor cells.^50^, ^99^ However, the number of such cells is limited, and even MSCs transplanted into the injury site rarely differentiate into oligodendrocyte‐like cells.^87^, ^92^

NT‐3 and TrkC activates intracellular signaling pathways that promote the survival and differentiation of oligodendrocyte precursor cells into myelin‐forming cells in the SC.^100^, ^101^, ^102^ GV‐EA increases NT‐3 levels in demyelinated areas of the SC and induces the differentiation of TrkC‐overexpressing MSCs pretreated with retinoic acid into oligodendrocyte‐like cells that form a myelin sheath around regenerating nerve fibers, and thereby restores nerve conduction pathways in the SC.^51^, ^52^, ^86^ These findings suggest a new strategy for the treatment of not only SCI, but also of demyelinating diseases such as multiple sclerosis.^103^ Table 1 summarizes the combination strategy of EA and adult stem cell transplantation in an animal model of SCI.

**TABLE 1:** A summary of the combination strategy of EA and adult tissue stem cell transplantation in an animal model of SCI

Strategies ^[Ref.]^	Species/Age and sex of animals	Cellular source of the stem cells	Neural cell differentiation of transplanted stem cells in the injury/graft site of spinal cord	Regenerating nerve fibers on in the injury/ graft site	Myelination in the injury/ graft site	Synapse‐like junction in the injury/graft site	Integration of grafted stem cells with host neural circuit in the injury/graft site	Clinical electrophysiological evaluation	Locomotion evaluation
EA+grafted NSCs^[^ ^77^ ^]^	Rat/Adult/Female	Rat hippocampus	Showing neuron‐like cells and astrocyte‐like cells	No nerve fiber regeneration shown	No myelination shown	No synapse‐like junction shown	No integration shown	No electrophysiological evaluation shown	No BBB score shown
EA+grafted MSCs^[^ ^87^ ^]^	Rat/Adult/Female	Rat hippocampus	Showing neuron‐like cells and oligodendrocyte‐like cells	Showing nerve fiber regeneration	Showing myelination	No synapse‐like junction shown	No integration shown	Showing strengthened SCEPs	Showing a higher score
EA+grafted BMSCs^[^ ^88^ ^]^	Rat/Adult/Male	Rat bone marrow	Showing neuron‐like cells and astrocyte‐like cells	No nerve fiber regeneration shown	No myelination shown	No synapse‐like junction shown	No integration shown	No electrophysiological evaluation shown	No BBB score shown
EA+grafted MSCs^[^ ^92^ ^]^	Rat/Adult/Female	Rat bone marrow	No cell differentiation was shown	Showing nerve fiber regeneration	No myelination shown	No synapse‐like junction shown	No integration shown	Showing strengthened MEPs	Showing a higher BBB score
EA+grafted BMSCs^[^ ^89^ ^]^	Rat/Adult/Male	Rat bone marrow	No cell differentiation was shown	No nerve fiber regeneration shown	No myelination shown	No synapse‐like junction shown	No integration shown	Showing strengthened SSEP	Showing a higher BBB score
EA+grafted MSCs^[^ ^91^ ^]^	Rat/Adult/Female	Rat bone marrow	Showing neuron‐like cells and oligodendrocyte‐like cells	Showing nerve fiber regeneration	No myelination shown	No synapse‐like junction shown	No integration shown	No electrophysiological evaluation shown	Showing a higher BBB score
EA+grafted TrkC‐MSCs^[^ ^48^ ^]^	Rat/Adult/Female	Rat bone marrow	Showing neuron‐like cells and oligodendrocyte‐like cells	Showing nerve fiber regeneration	Showing myelination	Showing synapse‐like junction	Showing the integration	Showing strengthened MEPs	Showing a higher BBB score
EA+grafted NR‐MSCs^[^ ^47^ ^]^	Rat/Adult/Female	Rat bone marrow	Showing neuron‐like cells and oligodendrocyte‐like cells	No nerve fiber regeneration shown	No myelination shown	No synapse‐like junction shown	No integration shown	No electrophysiological evaluation shown	No BBB score shown
EA+grafted TrkC‐MSCs^[^ ^51^ ^]^	Rat/Adult/Female	Rat bone marrow	Showing oligodendrocyte‐like cells	No nerve fiber regeneration shown	Showing myelination	No synapse‐like junction shown	No integration shown	Showing strengthened CMEP	Showing a lower error footsteps score
EA+grafted NR‐MSCs^[^ ^52^ ^]^	Rat/Female	Rat bone marrow	Showing oligodendrocyte‐like cells	No nerve fiber regeneration shown	Showing myelination	No synapse‐like junction shown	No integration shown	Showing strengthened CMEPs	No BBB score shown
EA+grafted SCED^[^ ^90^ ^]^	Dog/Adult/Male and female	Canine exfoliated deciduous teeth	No cell differentiation was shown	No nerve fiber regeneration shown	No myelination shown	No synapse‐like junction shown	No integration shown	No electrophysiological evaluation shown	Showing a higher functional evaluation score
EA+grafted NN^[^ ^53^ ^]^	Rat/Adult/Female	Rat hippocampus	Showing neurons, astrocytes, and oligodendrocytes	Showing nerve fiber regeneration	No myelination shown	Showing synapse‐like junction	Showing the integration	Showing strengthened CMEP and SSEP	Showing a higher BBB score
EA+grafted MN^[^ ^93^ ^]^	Rat/Adult/Female	Rat bone marrow	Showing neuron‐like cells	Showing nerve fiber regeneration	No myelination shown	Showing synapse‐like junction	Showing the integration	Showing strengthened CMEP	Showing a higher BBB score

Note

Abbreviations: BBB score, Basso, Beattie, and Bresnahan (BBB) open‐field locomotor test; BMSCs, bone marrow mesenchymal stromal cells; CMEP, cortical motor‐evoked potentials; EA, electro‐acupuncture; MEPs, motor‐evoked potentials; MN, TrkC gene‐modified bone marrow mesenchymal stem cell (TrkC‐MSC)‐derived neural network; MSCs, bone marrow mesenchymal stem cells; NN, NT‐3 and TrkC gene‐overexpressing neural stem cell (NSC)‐derived neural network; NR‐MSCs, neurotrophin‐3 (NT‐3) and retinoic acid (RA) preinduced bone marrow mesenchymal stem cells; NSCs, neural stem cells; SCED, stem cells from canine exfoliated deciduous teeth; SCEPs, spinal cord evoked potentials; SSEP, somatosensory evoked potentials; TrkC‐MSCs, tyrosine receptor kinase C (TrkC) gene‐modified bone marrow mesenchymal stem cells.

## 8 PERSPECTIVES

A large number of studies have investigated the applicability of GV‐EA in the treatment of SCI over the last two decades. Our research has revealed the mechanisms by which GV‐EA can be combined with adult stem cell transplantation to improve the microenvironment of the injured SC to promote the reconstruction of neuronal circuits. In this review, we discussed the multiple effects of GV‐EA, including stimulation of afferent nerve fibers of the meningeal branch of the SC for the transmission of information in the completely transected SC. We also described how GV‐EA can induce NT‐3 synthesis and secretion by cells in the injured SC and enhance the survival, differentiation, and migration of TrkC‐overexpressing NSCs and MSCs transplanted into the injury/graft site and their integration into existing neuronal circuits. We also describe how GV‐EA can assist in the replacement of injured host neurons, improve the microenvironment surrounding the injured tissue, stimulate nerve fiber regeneration and myelination, enhance cortical motor‐evoked potentials, and restore motor function in paralyzed limbs. The current evidence indicates that the combination of GV‐EA and gene‐modified adult stem cell transplantation is an innovative strategy for SCI treatment that warrants further development and testing in clinical settings (Figure 2).

Finally, recent advances in genetic engineering techniques and an improved knowledge of gene expression in adult stem cells have led to the demonstration that gene‐modified adult stem cells exhibit stronger therapeutic properties in some disease models compared with wild‐type adult stem cells. For example, a previous study investigated repair in a completely transected SCI and found that if wild‐type adult stem cells were transplanted directly, then the number of differentiated neurons would be smaller and they would not be able to effectively replace neurons that had died due to injury. Hence, gene‐modified adult stem cells may represent a useful option for transplantation to better differentiate into neurons and repair the neural circuits of the host SC. However, there remains an obvious gap between experimental models and clinical trials, along with a host of limitations related to the safety of gene‐modified adult stem cell transplantation for therapeutic use. It would be possible to avoid ethical issues if we were able to transplant another type of stem cell‐human induced pluripotent stem cells (hiPSCs). This is because the preparation of hiPSCs does not involve embryos and germ cells that are harvested from early humans.^104^, ^105^ More importantly, hiPSCs have unlimited capacity to expand; this could solve the problems associated with cell source and number, thus facilitating the advancement of clinical translational research. Studies have shown that the differentiation of hiPSC‐derived neural progenitor cells (NPCs) into neurons can be observed after the transplantation of NPCs into animals with a completely transected spinal cord.^106^ Differentiated neurons have been observed to develop long cellular processes to form synaptic connections with host neurons and promote the recovery of motor function after spinal cord injury.^106^, ^107^ Collectively, the available literature suggests whether it is possible to repair the structure and function of the injured spinal cord effectively if we apply the combination of electro‐acupuncture and the transplantation of hiPSC‐derived NPCs. More clinical studies are clearly desirable to ensure the safety profile of such treatment and to support the translation of this treatment model into a wide range of clinical settings. In addition, some researches indicated that sex differences exist a certain effect on the repair of central nervous system injury.^108^, ^109^, ^110^, ^111^, ^112^, ^113^, ^114^ Hence, future study should pay attention to the potential influence of sex differences on electro‐acupuncture and its combination with adult stem cell transplantation for spinal cord injury treatment.

## CONFLICT OF INTEREST

The authors declare no conflict of interest.

## AUTHOR CONTRIBUTIONS

Yuan‐shan Zeng was responsible for literature review, investigation, draft preparation and editing; Ying Ding was responsible for literature review and editing; Hao‐yu Xu was responsible for drawing and editing; Xiang Zeng, Bi‐Qin Lai, Ge Li, and Yuan‐Huan Ma were responsible for manuscript review. All authors have read and agreed to the published version of manuscript.

## Data Availability

Data sharing is not applicable to this article as no new data were created or analyzed in this study.
